# Repairing boundaries along pathways to tuberculosis case detection: a qualitative synthesis of intervention designs

**DOI:** 10.1186/s12961-021-00811-0

**Published:** 2022-01-10

**Authors:** Susanna S. van Wyk, Nancy Medley, Taryn Young, Sandy Oliver

**Affiliations:** 1 Centre for Evidence Based Health Care, Division of Epidemiology and Biostatistics, Department of Global Health, Stellenbosch University, Cape Town, South Africa; 2 Department of Clinical Sciences, Liverpool School of Tropical Medicine, Liverpool, United Kingdom; 3 EPPI-Centre, Social Science Research Unit, University College London, London, United Kingdom; 4 Faculty of the Humanities, University of Johannesburg, Johannesburg, South Africa

**Keywords:** Tuberculosis screening, Tuberculosis case finding, Tuberculosis case detection, Tuberculosis care pathways, Missing tuberculosis cases, Infectious disease screening, Logic model

## Abstract

**Background:**

Tuberculosis case-finding interventions often involve several activities to enhance patient pathways, and it is unclear which activity defines the type of case-finding intervention. When conducting studies to identify the most effective case-finding intervention it is important to have a clear understanding of these interventions for meaningful comparisons. This review aimed to construct a systems-based logic model of all pathways to tuberculosis case detection through a synthesis of intervention designs.

**Methods:**

We identified an existing systematic review on the effectiveness of interventions to increase tuberculosis case detection and updated the search from December 2016 to October 2020. We included randomized controlled trials, as these designs encourage detailed description of interventions. Taking each study in turn, intervention descriptions were read in detail. The texts were analysed qualitatively by constantly comparing emerging codes to construct patient journeys, visualized as logical chains. Actions taken as part of interventions were positioned along patient journeys to theorize the sequence of outcomes. Patient journeys formed the basis of the model, which was refined through discussion.

**Results:**

Based on intervention descriptions from 17 randomized controlled trials, our model distinguishes two care-seeking pathways and four screening pathways. An open invitation to people with tuberculosis symptoms creates care-seeking pathways. On care-seeking pathways, systematic screening can be conducted at general health services, but not at specific TB care services. People invited to tuberculosis services regardless of symptoms follow tuberculosis screening pathways and may be identified with presumptive tuberculosis even if they do not seek care for tuberculosis symptoms. Tuberculosis screening pathways include screening offered to all people accessing care at general health services, screening at a mobile clinic or health facility with open invitation to a whole population or tuberculosis contacts, screening personally offered to a whole population or tuberculosis contacts at home, work or school, and screening offered to people receiving care for human immunodeficiency virus or other clinical risk-group care.

**Conclusion:**

This systems-based logic model of tuberculosis case-finding pathways may support standardized terminology, consistency, transparency and improved communication among researchers, policy-makers, health workers and community members when implementing and evaluating interventions to improve tuberculosis case detection.

**Supplementary Information:**

The online version contains supplementary material available at 10.1186/s12961-021-00811-0.

## Background

Tuberculosis (TB) is a top infectious disease killer on our globe, with an estimated 1.4 million deaths in 2019. Even though TB is curable, transmission is ongoing and disease incidence is declining slowly, with a cumulative reduction of only 9% from 2015 to 2019 [1]. TB case detection is a key step in breaking this chain of transmission, but in countries where the burden of disease is highest, resources are limited and operational challenges in detecting missing cases are complex. As our understanding of interventions to decrease barriers and enhance pathways to TB case detection evolves, the terminology used to explain these interventions also evolves.

The current TB case detection framework depicts two pathways to case detection, namely the patient-initiated pathway and a complementary provider-initiated screening pathway [2, 3]. The term “patient-initiated pathway” was introduced in 2011 to improve the previously used term “passive case finding”, because the health system is not passive in its response to patients actively seeking care [2]. This pathway is initiated by a person experiencing and recognizing TB symptoms. It requires people to access an appropriate health facility where a health worker may identify them having presumptive TB and respond appropriately using a diagnostic algorithm to confirm diagnosis. After WHO abandoned “indiscriminate” mass screening in 1974 due to inefficiency of screening in low-burden populations and lack of basic diagnostic and treatment services in high-burden settings, interventions focused on enhancement of the patient-initiated pathway to TB case detection [4]. Screening interventions were offered mainly to TB contacts and people living with human immunodeficiency virus (PLHIV). However, in 2013 WHO revisited screening, and the screening pathway to TB case detection also became popular among other high TB-risk groups and populations [4, 5].

The screening pathway is described as a provider-initiated pathway, because the health system actively seeks out people with presumptive TB. “Systematic screening for active TB is defined as the systematic identification of people with suspected (presumptive) active TB, in a predetermined target group, using tests, examinations or other procedures that can be applied rapidly. Among those screened positive, the diagnosis needs to be established by one or several diagnostic tests and additional clinical assessments, which together have high accuracy” [5]. Screening may target different groups of people, can be applied in different settings and includes activities like health promotion and training of lay health workers [3, 5]. Definitions of TB screening interventions often reflect their different characteristics. Contact investigation describes screening of TB contacts, intensified case-finding (ICF) activities include screening of PLHIV, active case finding (ACF) is defined as systematic screening activities mostly outside health facilities, and passive case finding (PCF) with an element of systematic screening refers to screening of patients seeking care [3, 5–8]. Although “case finding” has been described as synonymous with “screening”, not all types of case-finding interventions are defined as screening interventions [9, 10]. For example, enhanced case finding (ECF) refers to TB information being made available to communities via health promotion activities to improve TB health-seeking behaviour and may or may not be combined with screening [5]. There is no clear distinguishing factor separating all the different case-finding interventions, and possible overlap exists. To detect TB along the patient-initiated pathway, the health system has to respond appropriately to patients actively seeking care, but exactly how a health system actively seeks TB patients and the appropriate response of people approached by a health system seeking TB patients is unclear. Screening pathways to case detection are depicted simply as pathways other than those initiated by the patient [3].

When conducting studies to identify the most effective, cost-effective, acceptable or feasible TB case-finding intervention it is important to have a clear understanding of these interventions for meaningful comparisons. This review aimed to construct a systems-based logic model of all pathways to TB case detection through a synthesis of intervention designs.

## Methods

The methods and findings of this review are reported in line with the ENTREQ (Enhancing transparency in reporting the synthesis of qualitative research) statement [11].

### Criteria for considering studies for this review

Any intervention aimed at improving early TB case detection at the community or primary healthcare level was included. Interventions aimed at improving TB case detection at the inpatient or laboratory level were excluded. Studies had to report on TB case detection as an outcome measure. The definition of a TB case was not restricted; however, latent TB infection (LTBI) and extrapulmonary TB were excluded. Only studies in countries with high TB burden, with an annual TB incidence of more than 10 cases per 100 000, were included [12]. Study design was restricted to individual or cluster-randomized controlled trials for analysis, where international reporting standards [13] guided by a checklist [14] encourage detailed description of interventions and sufficient information to deduce which pathway participants were following [15].

### Identification and selection of relevant studies

To avoid duplication of reviews, we first looked for existing reviews on this topic. We identified a Cochrane review aiming to evaluate the effectiveness of different strategies to increase TB case detection by improving access to TB diagnosis at primary healthcare or community-level services [16]. The authors included randomized and non-randomized controlled studies that assessed any intervention aimed at improving access to a TB diagnosis compared with no intervention or an alternative intervention. The review included the following comparisons: outreach TB screening with or without health promotion activities versus no intervention; health promotion activities versus no intervention; staff training compared to none; outreach TB screening versus health promotion; outreach clinic versus house-to-house screening; ACF interventions versus no intervention; and outreach TB services versus no intervention. The search was done in December 2016, and the review did not compare different types of screening interventions or different patient pathways.

As the search for studies for the review by Mhimbira et al. was done in December 2016, the same search was updated, using no language restrictions, in CENTRAL, MEDLINE and Embase from December 2016 to 6 October 2020. Search terms included “tuberculosis” and “case detection” or “case finding” or “systematic screening” (Additional file 1). Reference lists of included studies were searched.

Inclusion criteria were applied to the included studies in the Mhimbira review and studies identified by the updated search. One author (SVW) screened titles and abstracts. Full text articles of potential eligible studies were retrieved, and two authors (SVW and NM) assessed these articles for inclusion in the review. Discrepancies were resolved through discussion.

### Data collection and analysis

Our focus of interest was the reported descriptions of interventions to identify TB, rather than data to assess effectiveness of interventions. Taking each study in turn, working in alphabetical order of first author surnames, intervention descriptions were read in detail. The texts were analysed qualitatively by applying the constant comparative method, in which codes applied to each text were categorized and their properties and dimensions noted and “constantly compared… with all other parts of the data to explore variations, similarities and differences in data” [17]. The studies were not appraised for their quality, because our interest was in the intervention descriptions and not the assessment of effects. Quality assessment focuses on the methods of a study to identify imbalances between study groups. The quality of the methods could influence effect estimates, but it is unlikely that the quality of the methods would influence descriptions of the interventions.

Through constant comparative analysis of intervention descriptions, we constructed patient journeys that were visualized as logical chains or decision trees. Intervention activities, namely all reported actions taken as part of the intervention, were positioned along these various patient journeys to theorize the sequence of potential outcomes. Definitions used in our analysis and findings are reported in Table 1.

**Table 1:** Definitions used in the analysis and findings

Systematic screening for active TB	“The systematic identification of people with suspected (presumptive) active TB, in a predetermined target group, using tests, examinations or other procedures that can be applied rapidly. Among those screened positive, the diagnosis needs to be established by one or several diagnostic tests and additional clinical assessments, which together have high accuracy.” [5]
A screening tool	Tests, examinations or other procedures used for systematic screening for active TB. Examples of TB screening tools include a structured symptom-based questionnaire, chest radiography (CXR) or an algorithm [3]. Algorithms may include sequential or parallel tests. With sequential tests, only those who screen positive with the initial test receive a second test. With parallel tests, those who screen positive on any of the tests are regarded as positive screens
A diagnostic tool	Tests, examinations or other procedures used to establish a diagnosis of TB in people identified with presumptive TB. Examples of TB diagnostic tools include a clinical algorithm, sputum smear microscopy, Xpert MTB/RIF assay or culture [3]
TB symptom(s)	Any TB symptom, e.g. cough, fever, night sweats, weight loss or combination of TB symptoms as defined by the study authors
Care seeking	People seeking care for a perceived health problem
TB care seeking	People seeking care for TB symptoms specifically
A risk group	Any group of people in which the prevalence or incidence of TB is significantly higher than in the general population. Examples of risk groups include a whole population within a geographical area or TB contacts [5]
A clinical risk group	Individuals who are diagnosed with a specific disease or condition that increases their risk for TB, e.g. PLHIV
Presumptive TB	Identified when a provider identifies a patient with suspected active TB. In the context of screening, a person who screens positive is a presumptive TB case

In collaborative virtual meetings between the authors, we discussed the codes and evolving pathways, similar to methods employed for developing a systems-based logic model [18]. We tested the logic of these patient journeys by considering how each decision point might apply to people with different characteristics: with TB symptoms seeking care; with unrecognized TB symptoms; with preclinical active TB disease; with HIV. To develop the details of the logic model we also tested how decision points might apply to people when health workers use different screening tools and different diagnostic tools. This required theoretical sensitivity bearing in mind academic and clinical knowledge about working with marginalized groups. Thinking was further facilitated by early visualizations being refined by a professional designer to make clearer how the different pathways of the logic model were interrelated. Finally we compared pathways in the model to existing case-finding definitions.

## Results

### Identification of studies

**Fig. 1:**
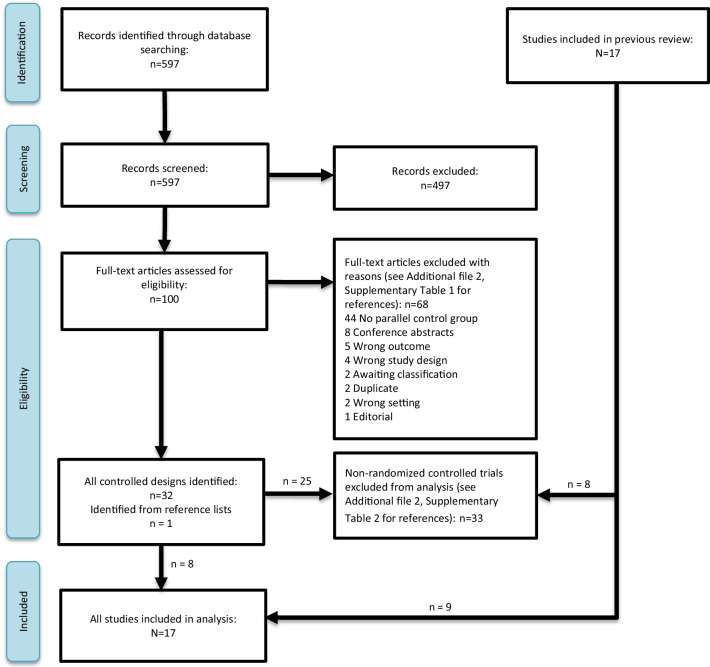
PRISMA [Preferred Reporting Items for Systematic Reviews and Meta-Analyses] flowchart

The Mhimbira review included nine randomized controlled trials and our updated search identified eight new randomized controlled trials of interventions to improve early TB case detection at the community or primary healthcare level in countries with high TB burden (Fig. 1).

### Description of studies

The details of excluded studies and characteristics of included studies are provided in Additional files 2 and 3: Table S1, respectively. Terminology used to describe interventions in included studies was inconsistent. It did not reflect whether an intervention could be classified as a screening intervention and could not be used to group similar interventions or distinguish dissimilar interventions (Additional file 3: Table S2).

### Logic model and pathways

Here we present (1) the overall systems-based logic model summarizing the pathways to TB case detection, and (2) a description of how the model was derived from the data and (3) how pathways differ from existing TB case-finding definitions.

**Fig. 2:**
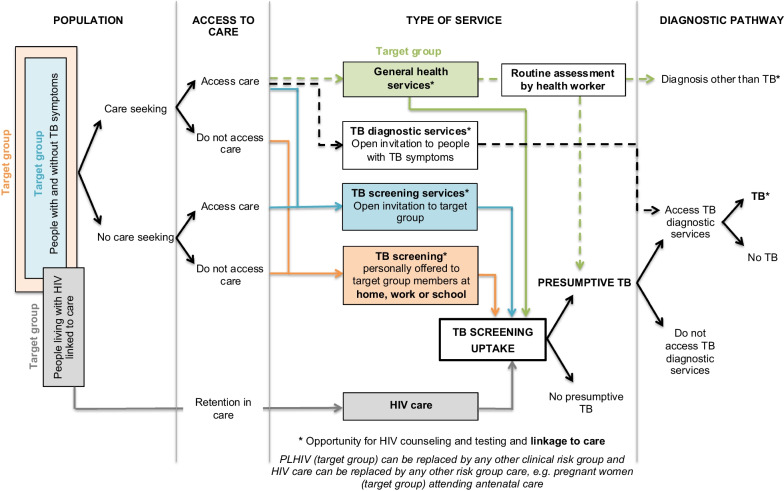
A systems-based logic model depicting types of services and associated pathways to TB case detection. The model distinguishes six pathways to TB case detection, namely two care-seeking pathways (green and black dashed lines) and four screening pathways (green, blue, orange and grey solid lines). People perceiving themselves to have a health problem and access general health services follow the general care-seeking pathway, where a provider can identify presumptive TB on routine assessment, i.e. history-taking and clinical examination, of an individual patient (green dashed line). People perceiving themselves to have TB symptoms may also follow the specific TB care-seeking pathway to TB diagnostic services, where all people accessing care are evaluated for possible active TB (black dashed line). People invited to TB services regardless of symptoms follow TB screening pathways and may be identified with presumptive TB even if they do not seek care for TB symptoms. Four screening pathways are distinguished: TB screening offered to all people accessing general health services (green solid line), dedicated TB screening services with open invitation to a whole population or TB contacts (blue solid line), TB screening offered to target group members at home, work or school (orange solid line) and TB screening offered to people living with HIV linked to care (grey solid line). A person who screens positive on the TB screening pathway is identified as a presumptive TB case and should receive confirmation of a diagnosis by accessing TB diagnostic services

**Fig. 3:**
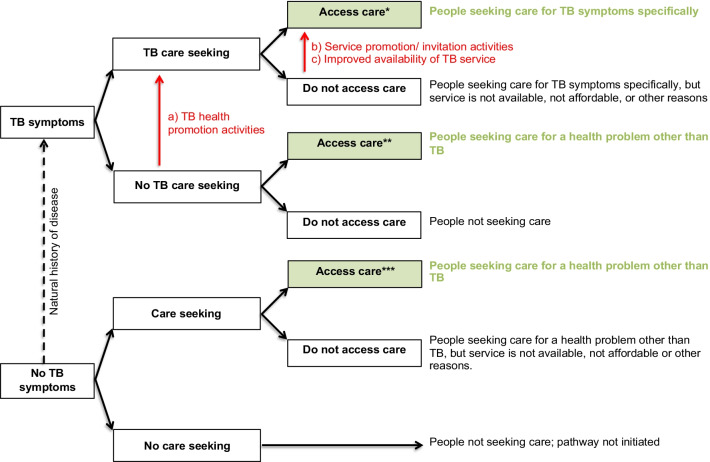
A decision tree to predict how activities (red) could influence TB care-seeking pathways. Starting with a group of people with and without TB symptoms and assuming people have to recognize their TB symptoms to seek care for TB symptoms (TB care seeking), this decision tree depicts how intervention activities (red text) could influence the group of people accessing care. At general health services (all green boxes), intervention activities may increase the number of people accessing care for TB symptoms specifically (*), but this group of people are mixed with people seeking care for health problems other than TB, among whom there may be people with unrecognized TB symptoms (**). For TB services exclusively inviting people with TB symptoms, the group of people accessing care only consists of people recognizing TB symptoms (*)

**Fig. 4:**
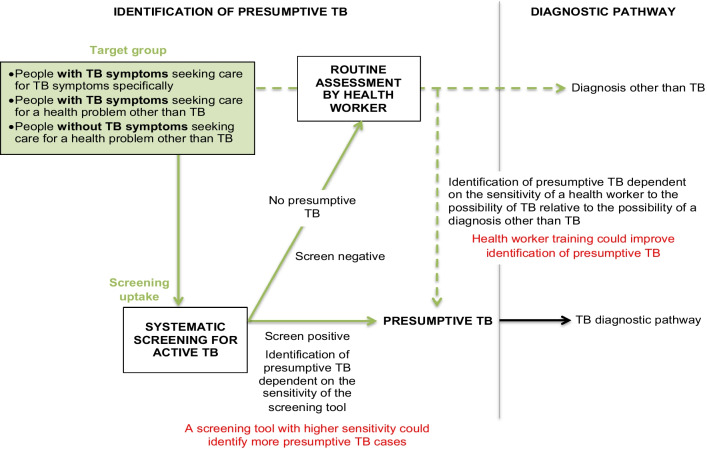
Identification of presumptive TB at general health services. The group accessing care at general health services (green box) may include people with TB symptoms seeking care for a health problem other than TB (refer to Fig. 3, green boxes). Identification of presumptive TB in these patients would be missed if a health worker assesses a patient and does not specifically ask about TB symptoms (green dashed line). TB screening systematically offered to all people accessing care, irrespective of their presenting complaint (green solid line), may identify people with presumptive TB who do not seek care for TB symptoms specifically. People with presumptive TB may access TB diagnostic services, while people who screen negative may follow routine assessment by a health worker

**Fig. 5:**
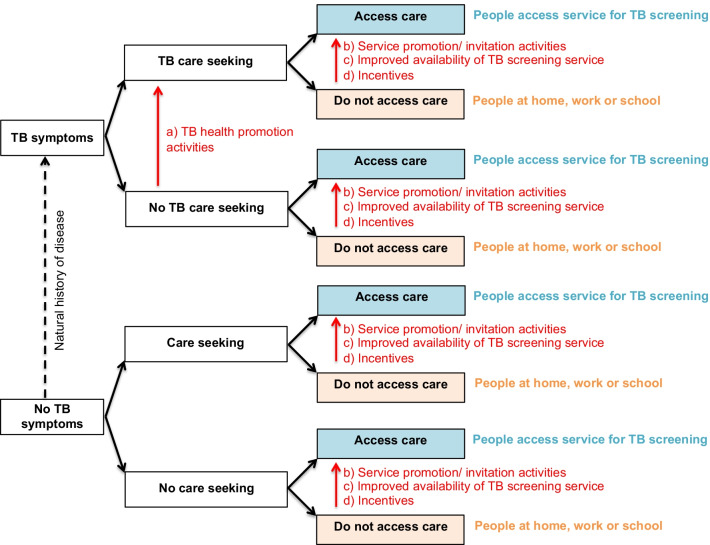
A decision tree to predict how activities (red) could influence pathways to dedicated TB screening. Starting with a group of people with and without TB symptoms, which represents a target group for screening, e.g. a whole population or TB contacts, this decision tree depicts how intervention activities (red text) could influence the group of people accessing TB screening. With an open invitation to a target group regardless of symptoms, the group accessing care (blue boxes) may consist of people with and without TB symptoms irrespective of whether they perceive themselves to have a health problem and irrespective of whether they seek care; however, TB health promotion may increase the proportion of people with TB symptoms within the group accessing care. By offering TB screening personally to people at their home, work or school, access barriers are bypassed (orange boxes)

**Fig. 6:**
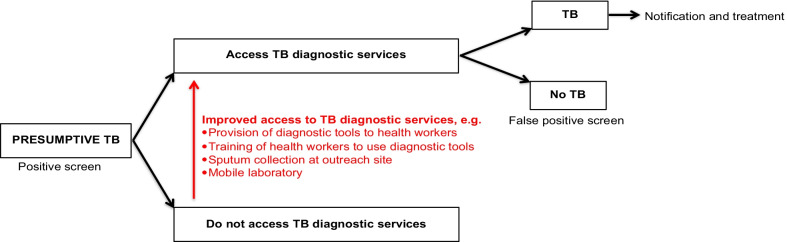
Pathway from presumptive TB to diagnosis, notification and treatment. This figure shows the section of the overall model after presumptive TB is identified. People identified with presumptive TB may be referred from a remote service to a central health facility for diagnosis. Access to TB diagnostic services can be improved by activities such as those depicted in red. The figure also shows that people identified with presumptive TB (positive screen) who do not have active TB (false-positive screen) will go through unnecessary diagnostic testing. A person diagnosed with active TB should be notified and started on treatment

1. The overall systems-based logic model
  Our model (Fig. 2) distinguishes six pathways to TB case detection, namely two care-seeking pathways and four screening pathways.
  Care-seeking pathways include the general care-seeking pathway and the TB care-seeking pathway. The general care-seeking pathway is initiated by patients experiencing and seeking care for any health problem, including TB symptoms, at general health services where health workers make individual diagnostic decisions based on routine assessment, namely history-taking and examination, of patients. The TB care-seeking pathway is initiated by TB services, at a health facility or mobile clinic, with open invitation to people with TB symptoms. The TB care-seeking pathway improves access to TB diagnostic services for people seeking care for TB symptoms specifically. Care-seeking pathways may miss people with active TB who do not seek care for TB symptoms, and these pathways can be enhanced with TB health promotion. On the general care-seeking pathway, TB case finding can be improved by systematic screening of all people accessing care, but on the specific TB care-seeking pathway, the group accessing care is self-selected and systematic screening is not feasible.
  TB screening pathways are initiated by services inviting people to TB screening regardless of symptoms. By inviting people regardless of symptoms, people without TB symptoms or symptoms not recognized as possibly due to TB could be identified with presumptive TB, depending on the sensitivity of the screening tool. Our model distinguishes four TB screening pathways: TB screening offered to all people accessing care at general health services, TB screening at a mobile clinic or health facility with open invitation to a whole population or TB contacts, TB screening personally offered to a whole population or TB contacts at home, work or school, and TB screening offered to people in HIV care or other clinical risk-group care.
  HIV counselling and testing added to TB screening at general health services or dedicated TB screening services may enhance pathways to HIV care. All pathways can be enhanced via improved access to TB diagnostic services, and all pathways can be enhanced via health system strengthening. Multiple strategies to enhance TB case detection may result in a combination of pathways to TB case detection within one community.
2. How the model was derived from the data
  Based on constant comparative analysis, intervention activities were coded into activity sets. All coded data with references are reported in Additional file 3: Table S3. Here we describe sections of the model and how each section was derived from these intervention activities.
  - Two care-seeking pathways *(Model section: Fig. **2**; dashed pathways)*
    Activities motivating people to attend a service include (a) TB health promotion activities, (b) service promotion/invitation activities and (c) activities to improve availability of a TB service, such as mobile TB service (Additional file 3: Table S3, Activity set 1). TB health promotion activities focusing on educating people about TB symptoms are assumed to raise TB awareness, to improve recognition of TB symptoms and to improve recognition on the importance of seeking care for TB symptoms. Service promotion or invitation activities and activities to improve availability of a TB service are assumed to improve access to a service. Based on these assumptions and starting with a group of people with and without TB symptoms, this decision tree predicts how these activities could influence peoples’ decision to access a service (Fig. 3). Two care-seeking pathways are distinguished: the care-seeking pathway to general health services, accessed by people seeking care for various health problems, including TB symptoms (Fig. 3; green boxes), and the TB care-seeking pathway to TB diagnostic services, accessed exclusively by people seeking care for TB symptoms (Fig. 3; *).
  - Care-seeking at general health services may include a screening pathway *(Model section: Fig. **2**; green pathways)*
    At general health services, people with TB symptoms may seek care for TB symptoms specifically or for a health problem other than TB (Fig. 4; green box). If a patient does not seek care for TB symptoms specifically and a health worker does not specifically ask about TB symptoms or consider the diagnosis of TB a strong possibility when examining a patient, identification of presumptive TB may be missed (Fig. 4; green dashed line). On this pathway, the identification of presumptive TB depends on the sensitivity of the health worker to the possibility of TB, relative to the possibility of a diagnosis other than TB. This sensitivity could be improved by training, logistical support and/or other activities to raise TB awareness among health workers (Additional file 3: Table S3, Activity 3a).
    Poor and inconsistent identification of presumptive TB by health workers may be bypassed by offering TB screening systematically to all patients accessing care, regardless of symptoms (Fig. 4, green solid line; also see Additional file 3: Table S3, Activity 3b). The more sensitive the screening tool, the more patients would screen positive and be identified with presumptive TB.
  - Two dedicated TB screening pathways *(Model section: Fig. **2**; blue and orange pathways)*
    Dedicated TB screening services invite all people in a target group (whole populations or TB contacts), regardless of symptoms. Two types of TB screening services can be distinguished, TB screening services at a clinic with open invitation to a target group and TB screening personally offered to target group members at their home, work or school.
    Activities motivating people to attend a screening service with open invitation include (a) TB health promotion activities, (b) service promotion or invitation activities, (c) activities to improve availability of a TB screening service, such as mobile TB screening service, and (d) incentives (Additional file 3: Table S3, Activity set 2A). These activities are added to the decision tree to depict how they could enhance the dedicated TB screening pathway (Fig. 5; red text). By inviting all people regardless of symptoms, the group accessing care consists of people with and without TB symptoms irrespective of whether they perceive themselves to have a health problem (Fig. 5; blue boxes); however, TB health promotion may increase the proportion of people with TB symptoms within the group accessing care.
    By offering TB screening personally to people at their home, work or school, access barriers are bypassed, further enhancing the dedicated TB screening pathway (Fig. 5; orange boxes). Activities to enhance TB screening uptake at these services include (a) TB health promotion activities and (b) repeat home visits (Additional file 3: Table S3, Activity set 2B).
  - TB screening offered to people living with HIV *(Model section: Fig. **2**; grey pathways)*
    TB screening is frequently offered to people living with HIV at HIV care services. TB screening at HIV care services could be enhanced by improved HIV diagnosis, linkage to care and retention in care. TB screening at general health services or at dedicated TB screening services provides the opportunity to add HIV counselling and testing with linkage to HIV care. See Additional file 3: Table S3, Activity set 5 for examples from included studies.
  - From identification of presumptive TB to diagnosis, notification and treatment *(Model section: Fig. **2**; pathways from presumptive TB to TB)*
    People identified with presumptive TB on any of the pathways should access TB diagnostic services for confirmation of a diagnosis (Fig. 6). If presumptive TB cases are identified at remote or informal services where diagnostic tools are not readily available, people are referred to a health facility for diagnosis. People seeking care for TB symptoms, who faced access barriers before identification of presumptive TB, may face the same access barriers when referred to a health facility. Activities to improve access to TB diagnostic services (Fig. 6, red text) include provision of diagnostic tools and training of health workers at remote and/or informal services, sputum collection in the community and setting up mobile laboratories. See Additional file 3: Table S3, Activity set 4 for examples from included studies.
  - Health system strengthening to support pathways
    Some activities were for strengthening the health system to improve TB case detection and to support the potential increase in TB case detection as a result of intervention activities. Health system strengthening is dependent on the established health system, the type of intervention, screening and diagnostic tools used, and planned steps for people who screen negative, people who screen false positive and people diagnosed with TB. Examples of health system strengthening include training of lay health workers to conduct screening [19], improved storage and transport of sputum specimens [20, 21], laboratory strengthening, health information system augmentation, and improved TB treatment and preventive therapy support [22]. Also see Additional file 3: Table S3, Activity set 6.
3. How the pathways differ from existing TB case-finding definitions
  WHO case-finding definitions have evolved over the development of successive guidelines. Here we look at some older definitions as well as more recent refinements to these definitions and how these definitions link to pathways in the model (Table 2). Our model distinguishes more pathways than existing definitions.

**Table 2:** Existing TB case-finding definitions and how they link to pathways in the model

Existing definitions		Pathways in the model
Passive TB case finding	Patient-initiated pathway to TB diagnosis involving (1) a person with active TB experiencing symptoms that he or she recognizes as serious; (2) the person having access to and seeking care, and presenting spontaneously at an appropriate health facility; (3) a health worker correctly assessing that the person fulfils the criteria for suspected TB; and (4) the successful use of a diagnostic algorithm with sufficient sensitivity and specificity to diagnose TB [3]	Our model distinguishes two types of care-seeking pathways. TB diagnostic services with open invitation to people with TB symptoms (Fig. 2, black dashed pathway) reflect the patient-initiated pathway. In our model it differs from the general care-seeking pathway, where patients with TB symptoms are mixed with people seeking care for other health problems (Fig. 2, green dashed pathway)
Passive case finding with an element of systematic screening	Passive case finding may involve an element of systematic screening if identification of people with suspected TB is done systematically for all people seeking care in a health facility or clinic [3]	At general health services, screening could be conducted to identify patients with presumptive TB who did not seek care for TB symptoms specifically. TB screening of all people accessing care at general health services represents PCF with an element of systematic screening (Fig. 2, green solid pathway). However systematic screening for active TB cannot be conducted at TB diagnostic services with open invitation to people with TB symptoms, as this group is self-selected
A triage test for TB	A test that can be rapidly conducted among people presenting to a health facility to differentiate those who should have further diagnostic evaluation for TB from those who should undergo further investigation for non-TB diagnoses [23]	In the model, a triage test for TB is the same as a screening test for TB in people seeking care at general health services
Enhanced TB case finding	Uses health information or education, or awareness campaigns to provide information about what type of health-seeking behaviour is appropriate when people experience symptoms of TB; this type of case finding may be combined with improving access to diagnostic services. Enhanced case finding may or may not be combined with screening [5, 23]	TB health promotion to improve TB care seeking along care-seeking pathways meets the definition of ECF. From our model, it is evident that the information provided via these health promotion messages may not be sufficient in enhancing screening pathways, which include non-care-seeking pathways. Careful consideration should be given to the content of health promotion messages when ECF is combined with screening, especially when screening is conducted with a tool that is sensitive enough to identify preclinical disease, e.g. CXR. In this context, health information or education about risk and prevention of TB can enhance non-care-seeking pathways
Active TB case finding	Synonymous with systematic screening for active TB, although it normally implies screening that is implemented outside of health facilities [3] This definition has recently been refined to the following: provider-initiated screening and testing in communities by mobile teams, often using mobile X-ray and rapid molecular tests. The term is sometimes used synonymously with systematic screening [23]	Dedicated TB screening services (Fig. 2, blue and orange pathways) reflect ACF. The target group could include a whole population or TB contacts (see TB contact investigation). Our model distinguishes between two types of dedicated TB screening services. The main distinguishing factor separating the two types is the availability of screening TB screening personally offered to target group members at home, work or school (Fig. 2, orange pathway) bypasses access barriers and enhances the dedicated TB screening pathway
TB contact investigation	A systematic process for identifying previously undiagnosed people with TB disease and TB infection among the contacts of an index TB patient and/or other comparable settings where transmission occurs. Contact investigation consists of identification, clinical evaluation and/or testing and provision of appropriate anti-TB therapy (for people with confirmed TB) or TB preventive treatment (for those without TB disease) [8]	TB contact investigation does not imply a specific type of case-finding intervention. TB contacts may be invited to general health services or TB diagnostic services if they develop TB symptoms (care-seeking pathways). TB contacts may also be invited to dedicated TB screening services at a health facility or at their home (Fig. 2, blue and orange pathways)
Intensified TB case finding	WHO guidance for intensified TB case finding states, “All people living with HIV, wherever they receive care, should be regularly screened for TB using a clinical algorithm at every visit to a health facility or contact with a health worker.” [6]	Intensified case finding is represented by screening of PLHIV linked to care (Fig. 2, grey pathway). Of note, “HIV” can be replaced by any other “clinical risk group” and “HIV care” can be replaced by any other “risk group care”, e.g. screening of pregnant women linked to antenatal care

## Discussion

### Summary of findings

Through a synthesis of intervention designs, we constructed a systems-based logic model distinguishing the following case-finding pathways to TB case detection: two care-seeking pathways (seeking general healthcare or specific TB care), and four provider-initiated pathways: (a) screening offered to all people accessing care at general health services, (b) TB screening at a mobile clinic or health facility with open invitation to a whole population or TB contacts, (c) TB screening personally offered to a whole population or TB contacts at home, work or school, and (d) TB screening offered to people in HIV care or other clinical risk group care.

### Potential benefits of the new systems-based logic model

The new model has several benefits. It aligns asymptomatic and non-TB care-seeking pathways with the availability of TB screening services. By including these pathways, the model depicts the reality of people with and without TB symptoms, moving in parallel from seeking care to accessing care. It provides a way to visualize potential relative differences in numbers needed to screen and costs depending on the design of the screening programme, and therefore supports comparison of cost-effectiveness between different types of screening interventions. The model was developed to include multiple screening and/or diagnostic tools rather than to be tool-specific and, as another benefit, creates the opportunity to evaluate diagnostic test accuracy of different screening tools within a specific type of screening intervention**.** When evaluating acceptability of screening programmes, the model provides a way to predict possible benefits and harms for people along the pathway to TB case detection. It also depicts potential barriers for poor uptake of TB screening and could be used as a tool to support monitoring and evaluation of TB screening interventions.

Another important consideration when thinking about screening interventions is equity. Inconsistent identification of a high-risk group may result in inequitable access to TB screening. A clear objective definition of a high-risk target group, irrespective of TB symptoms, supports offering of screening to all people eligible for screening, for example all people within a geographical boundary, all people accessing care or all household contacts of a TB index case. Such a target group, irrespective of TB symptoms, is also depicted in our model. If a high-risk group is an unidentified subgroup of a larger population, such as smokers, the larger population can be screened with an initial question about smoking habits to identify all people eligible for further TB screening.

### How the new model relates to wider literature

In the literature, many models have been used to explain and quantify gaps in TB case detection. The onion model is probably the most well known and is used to visualize the fraction of all TB cases that are diagnosed and reported to national TB programmes (NTP) [2]. The outer layers of the onion represent people who are not diagnosed, the more inner layers represent people who are diagnosed but not reported to the NTP, and the innermost layer represents people who are diagnosed and reported to the NTP. Bassilli et al. linked programme indicators to the onion model, with different layers representing different types of health facilities and the outer layer representing undetected cases in the community [24]. Wells et al. updated this model in 2017 to show possible interventions that could be conducted by NTPs to find missing cases as patients move from the outside to the inside of the onion [25]. This model does not represent people at health facilities who do not seek care for TB symptoms but who may have active TB.

Another model is the care cascade [26, 27]. Care cascades are used to show gaps in the health system where TB patients drop out of care before completing TB treatment. It provides a way to monitor TB programmes and target interventions to bridge gaps and ensure a continuum of care. The first gap in the care cascade is the case-finding gap [28]. Interventions proposed to bridge this gap include ACF in the community, ACF at health facilities, ACF of high-risk groups or ACF at private facilities. But the care cascade does not explain differences between these ACF strategies and how one strategy would miss cases found by another strategy.

Patient pathway analysis is used as a complementary approach to care cascades to align the availability of TB services to where patients seek care [26]. However, these pathways only include patients seeking care for TB symptoms.

None of these models has aligned asymptomatic and non-TB care-seeking pathways with the availability of TB screening services. By including these pathways, the new model depicts the reality of people with and without TB symptoms, moving in parallel from seeking care to accessing care.

If screening interventions are directly applied to the TB patient-initiated pathway without taking parallel asymptomatic and other care-seeking pathways into account, “systematic screening” may be misinterpreted and the benefit of screening may be missed. The TB patient-initiated pathway is depicted as a linear pathway starting with a patient exposed to TB and experiencing TB symptoms, to accessing care at a health facility [3]. People who do not seek care for TB symptoms do not reach a health facility along this impaired pathway, while the benefit of screening is exactly to identify those TB cases not seeking care for their TB symptoms. Our review showed that TB services offered only to people seeking care for TB symptoms are sometimes labelled “screening” or “ACF” services if such services are provided outside a health facility (Additional file 3: Table S2). If these studies are included as ACF interventions in comparisons when analysing effectiveness of TB screening [16], the true effectiveness of TB screening may be underestimated. Future research on effectiveness of different case-finding strategies may consider using the different pathways from our model.

## Strengths and limitations of the study

Our study has several strengths. Methods were transparent and combined the strengths of systematic synthesis with team development of a logic model. The diverse backgrounds of team members supported interpretation of the interaction between people, providers and the health system. Relevant team backgrounds include medicine, social sciences and epidemiology, with experience in clinical and primary research work in settings of high TB and HIV burden, as well as experience in systematic reviews, qualitative evidence synthesis and diagnostic and screening methodology.

Our study has some limitations. Interventions targeting high-risk groups such as prisoners or refugees were not included in the analysis. However, the model is generic, and a target group with and without TB symptoms can represent all people within a residential institution, such as prisons, or all refugees within a camp. Only one reviewer screened titles and abstracts, and this may have introduced selection bias.

Furthermore, intervention activities were not always clearly described in all trials, and some interventions could have been misunderstood and misclassified. As we did not look at effectiveness as an outcome, bias due to misclassification is negligible. On the contrary, inconsistent reporting of intervention activities could be improved by using the new model in the future.

Lastly, data on TB preventive therapy and TB treatment support and notification were not presented. This was due to two factors: (1) these data were not always reported and (2) our intention was to focus specifically on the knowledge gap concerning the first part of the TB care cascade and not the full pathway to treatment, notification and cure.

## Conclusion

The systems-based logic model of two care-seeking pathways and four TB screening pathways may support standardized terminology, consistency, transparency and improved communication among researchers, policy-makers, health workers and community members when implementing, monitoring and evaluating interventions to find missing cases and improve TB case detection.

## Supplementary Information

**Additional file 1.** Search terms. Terms used to search in Medline, Embase and The Cochrane Library.

**Additional file 2.** Excluded studies. **Table S1.** A list of excluded studies according to reason for exclusion. **Table S2.** A list of non-randomized controlled trials excluded. References of excluded studies.

**Additional file 3.** Data tables. **Table S1.** Characteristics of included studies. **Table S2.** Authors’ description of interventions. **Table S3.** Intervention activities (codes), References.

## Data Availability

All data generated or analysed during this study are included in this published article and its additional information files.

## Abbreviations

ACF: Active case finding
CXR: Chest radiography
ECF: Enhanced case finding
HIV: Human immunodeficiency virus
ICF: Intensified case finding
LTBI: Latent tuberculosis infection
NTP: National tuberculosis programme
PCF: Passive case finding
PLHIV: People living with human immunodeficiency virus
TB: Tuberculosis
