# Supplementary material for: Repairing boundaries along pathways to tuberculosis case detection: a qualitative synthesis of intervention designs
Source: Health Res Policy Syst. 2022 Jan 10;20:7. doi: 10.1186/s12961-021-00811-0 (PMC8751340; doi:10.1186/s12961-021-00811-0)
Supplement: Supplementary file 1 — Additional file 1. Search terms. Terms used to search in Medline, Embase and The Cochrane Library. [file 12961_2021_811_MOESM1_ESM.pdf]

Medline

1	tuberculosis [MeSH]
2	tuberculosis [ti, ab]
3	Mycobacterium tuberculosis [MeSH]
4	Case* detection ti, ab
5	Case* finding ti, ab
6	Systematic screening* ti, ab
7	1 or 2 or 3
8	4 or 5 or 6
9	Diagnos* OR detect* OR screen* OR assess* ti, ab
10	7 and 8 and 9

Embase

1	Tuberculosis [Emtree]
2	Tuberculosis [ti, ab]
3	Mycobacterium tuberculosis [Emtree]
4	Case* detection ti, ab
5	Case* finding ti, ab
6	Systematic screening* ti, ab
7	Case finding [Emtree]
8	1 or 2 or 3
9	4 or 5 or 6 or 7
10	Diagnos* OR detect* OR screen* OR assess* ti, ab
11	8 and 9 and 10

The Cochrane Library

#1 tuberculosis
#2 MeSH descriptor: [Tuberculosis] explode all trees
#3 MeSH descriptor: [Mycobacterium tuberculosis] explode all trees
#4 #1 or #2 or #3
#5 “case detection” or “case finding” or “systematic screening”
#6 #4 and #5
