# Supplementary material for: Repairing boundaries along pathways to tuberculosis case detection: a qualitative synthesis of intervention designs
Source: Health Res Policy Syst. 2022 Jan 10;20:7. doi: 10.1186/s12961-021-00811-0 (PMC8751340; doi:10.1186/s12961-021-00811-0)
Supplement: Supplementary file 2 — Additional file 2. Excluded studies. Table S1. A list of excluded studies according to reason for exclusion. Table S2. A list of non-randomized controlled trials excluded. References of excluded studies. [file 12961_2021_811_MOESM2_ESM.pdf]

## Table S1: List of excluded studies according to reason for exclusion

| No parallel control group (n = 44) | Conference abstracts (n = 8) | Wrong outcome (n = 5) | Wrong study design (n = 4) | Awaiting classification (n = 2) | Duplicate (n = 2) | Wrong setting (n = 2) | Editorial (n = 1) |
|---|---|---|---|---|---|---|---|
| Andre 2018 (1) | Izco 2019 (2) | Auld 2020 (3) | Shah 2017 (4) | Chatla 2018 (5) | Agizew 2019 (6) | Adane 2019 (7) | Dheda 2018 (8) |
| Aye 2018 (9) | Kunwipakorn 2019 (10) | Awan 2018 (11) | Shewade 2019 (12) | Khatana 2017 (13) | Fox 2017 (14) | Kuehne 2018 (15) | |
| Balcells 2020 (16) | Lee 2019 (17) | Blanc 2018 (18) | Shukla 2020 (19) | | | | |
| Beyanga 2018 (20) | Merle 2017 (21) | McHembere 2019 (22) | Teo 2020 (23) | | | | |
| Bigogo 2018 (24) | Mirembe 2019 (25) | Page-Shipp 2018 (26) | | | | | |
| Bosa 2017 (27) | Muhoozi 2020 (28) | | | | | | |
| Burmen 2018 (29) | Nliwasa 2018 (30) | | | | | | |
| Camelique 2019 (31) | Phuong 2019 (32) | | | | | | |
| Chen 2017 (33) | | | | | | | |
| Choun 2019 (34) | | | | | | | |
| Codlin 2018 (35) | | | | | | | |
| Habib 2020 (36) | | | | | | | |
| Hemavarneshwari 2019 (37) | | | | | | | |
| Islam 2017 (38) | | | | | | | |
| Joshi 2017 (39) | | | | | | | |
| Kamenska 2019 (40) | | | | | | | |
| Karamagi 2018 (41) | | | | | | | |
| Karki 2017 (42) | | | | | | | |
| Kigozi 2019 (43) | | | | | | | |
| Kim 2019 (44) | | | | | | | |
| Koura 2017 (45) | | | | | | | |
| Kyaw 2019 (46) | | | | | | | |
| Lee 2019 (47) | | | | | | | |
| Liu 2019 (48) | | | | | | | |
| Madhani 2020 (49) | | | | | | | |
| Mandalakas 2017 (50) | | | | | | | |
| Martinez 2018 (51) | | | | | | | |
| Maung 2017 (52) | | | | | | | |
| Mekonnen 2018 (53) | | | | | | | |
| Moh 2017 (54) | | | | | | | |
| Munim 2020 (55) | | | | | | | |
| Myint 2017 (56) | | | | | | | |
| Naowarat 2019 (57) | | | | | | | |
| Ohene 2018 (58) | | | | | | | |
| Okelloh 2019 (59) | | | | | | | |
| Oshi 2017 (60) | | | | | | | |

| | | | | | | | |
|---|---|---|---|---|---|---|---|
| Putra 2019 (61) | | | | | | | |
| Sander 2019 (62) | | | | | | | |
| Shenoi 2017 (63) | | | | | | | |
| Soe 2017 (64) | | | | | | | |
| Tefera 2019 (65) | | | | | | | |
| Ullah 2020 (66) | | | | | | | |
| Zawedde-Muyanja 2018 (67) | | | | | | | |
| Zhang 2019 (68) | | | | | | | |

## Table S2: List of non-randomised controlled trials excluded (n = 33)

| |
|---|
| Abdullahi 2020 (69) |
| Bogdanova 2019 (70) |
| Chen 2019 (71) |
| Daftary 2019 (72) |
| Datiko 2017 (73) |
| Datta 2019 (74) |
| Gurung 2019 (75) |
| Hussain 2019 (76) |
| James 2017 (77) |
| Jaramillo 2001 (78) |
| Jose 2020 (79) |
| Joshi 2015 (80) |
| Khan 2012 (81) |
| Khan 2016 (82) |
| Khatana 2019 (83) |
| Lung 2019 (84) |
| Malik 2018 (85) |
| McAllister 2017 (86) |
| Morishita 2016 (87) |
| Morishita 2017 (88) |
| Ohene 2017 (89) |
| Oshi 2016 (90) |
| Pan 2018 (91) |
| Reddy 2015 (92) |
| Saunders 2019 (93) |
| Shah 2020 (94) |
| Shewade 2019 (95) |
| Ustero 2017 (96) |
| Vo 2020 (97) |
| Vyas 2019 (98) |

| | |
|---|---|
| Yassin 2013 (99) | |
| Yellappa 2018 (100) | |
| Yuen 2019 (101) | |

## References (excluded studies)


1. Andre E, Rusumba O, Evans CA, Ngongo P, Sanduku P, Elvis MM, et al. Patient-led active tuberculosis case-finding in the Democratic Republic of the Congo. Bull World Health Organ. 2018;96(8):522–30.
2. Izco S, Murias A, Jordan A, Greene G, Catorze N, Lucinda X, et al. Diagnosing tuberculosis in people living with HIV in a rural district of Mozambique: Yield of TB-LAM, digital chest X ray and XpertUltra. HIV Med. 2019;20 Suppl 9:31.
3. Auld AF, Agizew T, Mathoma A, Boyd R, Date A, Pals SL, et al. Effect of tuberculosis screening and retention interventions on early antiretroviral therapy mortality in Botswana: a stepped-wedge cluster randomized trial. Bmc Med. 2020;18(1):19.
4. Shah L, Rojas M, Mori O, Zamudio C, Kaufman JS, Otero L, et al. Cost-effectiveness of active case-finding of household contacts of pulmonary tuberculosis patients in a low HIV, tuberculosis-endemic urban area of Lima, Peru. Epidemiol Infect. 2017;145(6):1107–17.
5. Chatla C, Jaju J, Achanta S, Samyuktha R, Chakramahanti S, Purad C, et al. Active case finding of rifampicin sensitive and resistant TB among household contacts of drug resistant TB patients in Andhra Pradesh and Telangana states of India - A systematic screening intervention. Indian J Tuberc. 2018;65(3):218–24.
6. Agizew T, Chihota V, Nyirenda S, Tedla Z, Auld AF, Mathebula U, et al. Tuberculosis treatment outcomes among people living with HIV diagnosed using Xpert MTB/RIF versus sputum-smear microscopy in Botswana: a stepped-wedge cluster randomised trial. BMC Infect Dis. 2019;19(1):1058.
7. Adane K, Spigt M, Winkens B, Dinant GJ. Tuberculosis case detection by trained inmate peer educators in a resource-limited prison setting in Ethiopia: a cluster-randomised trial. Lancet Glob Heal. 2019;7(4):e482–91.
8. Dheda K. “Mind the gap”: detecting the missing TB cases through active case finding. Int J Tuberc Lung Dis. 2018;22(10):1115.
9. Aye S, Majumdar SS, Oo MM, Tripathy JP, Satyanarayana S, Kyaw NTT, et al. Evaluation of a tuberculosis active case finding project in peri-urban areas, Myanmar: 2014-2016. Int J Infect Dis. 2018;70:93–100.
10. Kunwipakorn K, Chierakul N. Community active case finding for pulmonary tuberculosis. Respirology. 2019;24 Suppl 2:30.
11. Awan WM, Zaidi SMA, Habib SS, Khowaja S, Malik A, Khan U, et al. Impact of scaling up Xpert R MTB/RIF testing for the detection of rifampicin-resistant TB cases in Karachi, Pakistan. Int J Tuberc Lung Dis. 2018;22(8):899–904.
12. Shewade HD, Gupta V, Satyanarayana S, Kumar S, Pandey P, Bajpai UN, et al. Active versus passive case finding for tuberculosis in

marginalised and vulnerable populations in India: comparison of treatment outcomes. Glob Health Action. 2019;12(1):1656451.
13. Khatana GH, Masoodi M, Amin M. Factors affecting applicability of "home-based interventional model" for active case finding among household contacts of index cases of pulmonary tuberculosis in Kashmir. Indian J Tuberc. 2017;64(3):189–95.
14. Fox G, Nhung N V, Ho LTN, Anh NT, Hoa NB, Buu TN, et al. A cluster randomised controlled trial of contact investigation for the detection of tuberculosis: The ACT2 trial. Respirology. 2017;22 Suppl 3:61.
15. Kuehne A, Hauer B, Brodhun B, Haas W, Fiebig L. Find and treat or find and lose? Tuberculosis treatment outcomes among screened newly arrived asylum seekers in Germany 2002 to 2014. Euro Surveill. 2018;23(11).
16. Balcells ME, Carvajal C, Fernandez P, Ruiz-Tagle C, Pizarro A, Garcia P, et al. [Systematic screening of adult household contacts of patients with active pulmonary tuberculosis reveals high rates of new infections during follow-up]. Estrategia de pesquisa sistemática seguimento prolongado revela alto número de nuevas infecciones tuberculosas en contactos adultos en la Región Metropolitana Chile. Rev Med Chile. 2020;148(2):151–9.
17. Lee S, Lau L, Lim K, Cole D. Incentives that influence low income Filipinos with Tuberculosis symptoms to change health-seeking behaviour: a randomized controlled trial. Int J Infect Dis. 2019;79 Suppl 1:11.
18. Blanc FX, Badje AD, Bonnet M, Gabillard D, Messou E, Muzoora C, et al. Systematic vs test-guided tuberculosis treatment: data of the statis randomized trial. Top Antivir Med. 2018;26:13s-14s.
19. Shukla G, Sarin R, Vohra V, Khayyam KU. To study the impact of active case finding (ACF) among the TB patients detected in South Delhi. Indian J Tuberc. 2021;68(1):80-4.
20. Beyanga M, Kidenya BR, Gerwing-Adima L, Ochodo E, Mshana SE, Kasang C. Investigation of household contacts of pulmonary tuberculosis patients increases case detection in Mwanza City, Tanzania. BMC Infect Dis. 2018;18(1):110.
21. Merle CS, Bonsu FA, Sarr M, Adusi-Poku Y, Toure N, Ndiaye JL, et al. Integrated approaches for intensifying tuberculosis active case finding: Lessons learned from pilot projects in West Africa. Trop Med Int Heal. 2017;22 Suppl 1:215.
22. McHembere W, Agaya J, Yuen CM, Okelloh D, Achola M, Opole J, et al. High yield of new HIV diagnoses during active case-finding for tuberculosis. AIDS. 2019;33(15):2431-5.
23. Teo AKJ, Prem K, Tuot S, Ork C, Eng S, Pande T, et al. Mobilising community networks for early identification of tuberculosis and treatment initiation in Cambodia: an evaluation of a seed-and-recruit model. ERJ open Res. 2020;6(2).
24. Bigogo G, Cain K, Nyole D, Masyongo G, Auko JA, Wamola N, et al. Tuberculosis case finding using population-based disease surveillance platforms in urban and rural Kenya. BMC Infect Dis. 2018;18(1):262.
25. Mirembe J, Kyomugisha B, Mugadu J, Nkurunziza P, Mugume N, Amutuhaire W. Strengthening tuberculosis diagnosis and notification through tb surge: Experience of Uganda protestant medical bureau. Trans R Soc Trop Med Hyg. 2019;113 Suppl

1:S273–4.
26. Page-Shipp L, Lewis JJ, Velen K, Senoge S, Zishiri E, Popane F, et al. Household point of care CD4 testing and isoniazid preventive therapy initiation in a household TB contact tracing programme in two districts of South Africa. PLoS One. 2018;13(3):e0192089.
27. Bosa L, Da Silva L, Mendes D V, Sifna A, Sargento Mendes M, Riccardi F, et al. Feasibility and Effectiveness of Tuberculosis Active Case-Finding among Children Living with Tuberculosis Relatives: a Cross-Sectional Study in Guinea-Bissau. Mediterr J Hematol Infect Dis. 2017;9(1):e2017059.
28. Muhoozi M, Tusabe J, Tabwenda L, Jimale SM. Pin19 a Cost Effectiveness Analysis of Active Case Finding and Passive Case Finding for Case Detection of Tuberculosis at Kisugu Hc Iii,Kampala Uganda. Value Heal. 2020;23 Suppl 1:S171–2.
29. Burmen BK, Mogunde J, Malika T. The Use of Laypersons to Support Tuberculosis Screening at a Kenyan Referral Hospital. Int J Prev Med. 2018;9:44.
30. Nliwasa M, Choko A, Mwapasa M, Grint D, MacPherson P, Corbett EL. Trends in HIV- and sex-stratified tuberculosis case notifications in Blantyre, Malawi. Top Antivir Med. 2018;26 Suppl 1:340s.
31. Camelique O, Scholtissen S, Dousset JP, Bonnet M, Bastard M, Hewison C. Mobile community-based active case-finding for tuberculosis among older populations in rural Cambodia. Int J Tuberc Lung Dis. 2019;23(10):1107–14.
32. Phuong NTB, Anh NT, Khanh LB, Tu Oanh LT, Van Son N, Hoa NB, et al. The role of digital chest radiography and genexpert MTB/RIF in community-wide active case finding for tuberculosis. Respirology. 2019;24 Suppl 2:30–1.
33. Chen C, Yang CG, Gao X, Lu ZZ, Tang FX, Cheng J, et al. Community-based active case finding for tuberculosis in rural western China: a cross-sectional study. Int J Tuberc Lung Dis. 2017;21(11):1134–9.
34. Choun K, Decroo T, Mao TE, Lorent N, Gerstel L, Creswell J, et al. Performance of algorithms for tuberculosis active case finding in underserved high-prevalence settings in Cambodia: a cross-sectional study. Glob Health Action. 2019;12(1):1646024.
35. Codlin AJ, Monyrath C, Ky M, Gerstel L, Creswell J, Eang MT. Results from a roving, active case finding initiative to improve tuberculosis detection among older people in rural cambodia using the Xpert MTB/RIF assay and chest X-ray. J Clin Tuberc other Mycobact Dis. 2018;13:22–7.
36. Habib SS, Rafiq S, Jamal WZ, Ayub SM, Ferrand RA, Khan A, et al. Engagement of private healthcare providers for case finding of tuberculosis and diabetes mellitus in Pakistan. BMC Health Serv Res. 2020;20(1):328.
37. Hemavarneshwari S, Shaikh RB, Naik PR, Nagaraja SB. Strategy to sensitize private practitioners on RNTCP through medico-social workers in urban field practice area of a Medical College in Bengaluru, Karnataka. Indian J Tuberc. 2019;66(2):253–8.
38. Islam Z, Sanin KI, Ahmed T. Improving case detection of tuberculosis among children in Bangladesh: lessons learned through an implementation research. BMC Public Health. 2017;17(1):131.

39. Joshi D, Sthapit R, Brouwer M. Peer-led active tuberculosis case-finding among people living with HIV: lessons from Nepal. Bull World Health Organ. 2017;95(2):135–9.
40. Kamenska N, Nabirova D, Davtyan K, Davtyan H, Zachariah R, Aslanyan G. Strategies for active detection of tuberculosis in Ukraine: Comparative effectiveness amongst key populations (2014-2018). J Infect Dev Ctries. 2019;13(7.1):89S-94S.
41. Karamagi E, Sensalire S, Muhire M, Kisamba H, Byabagambi J, Rahimzai M, et al. Improving TB case notification in northern Uganda: evidence of a quality improvement-guided active case finding intervention. BMC Health Serv Res. 2018;18(1):954.
42. Karki B, Kittel G, Bolokon Jr. I, Duke T. Active Community-Based Case Finding for Tuberculosis With Limited Resources. Asia-Pacific J Public Heal. 2017;29(1):17–27.
43. Kigozi NG, Heunis JC, Engelbrecht MC. Yield of systematic household contact investigation for tuberculosis in a high-burden metropolitan district of South Africa. BMC Public Health. 2019;19(1):867.
44. Kim H, Kim HJ, Oh KH, Oh HW, Choi H. A Pilot Project of Systematic Tuberculosis Screening in the Elderly in a South Korean Province. Tuberc Respir Dis. 2019;82(3):194–200.
45. Koura KG, Trebucq A, Schwoebel V. Do active case-finding projects increase the number of tuberculosis cases notified at national level? Int J Tuberc Lung Dis. 2017;21(1):73-78.
46. Kyaw NTT, Sithu A, Satyanarayana S, Kumar AM V, Thein S, Thi AM, et al. Outcomes of Community-Based Systematic Screening of Household Contacts of Patients with Multidrug-Resistant Tuberculosis in Myanmar. Trop Med Infect Dis. 2019;5(1).
47. Lee S, Lau L, Lim K, Ferma J, Dodd W, Cole D. The Presence of Cough and Tuberculosis: Active Case Finding Outcomes in the Philippines. Tuberc Res Treat. 2019;2019:4578329.
48. Liu K, Peng Y, Zhou Q, Cheng J, Yu H, Tang L, et al. Assessment of active tuberculosis findings in the eastern area of China: A 3-year sequential screening study. Int J Infect Dis. 2019;88:34–40.
49. Madhani F, Maniar RA, Burfat A, Ahmed M, Farooq S, Sabir A, et al. Automated chest radiography and mass systematic screening for tuberculosis. Int J Tuberc Lung Dis. 2020;24(7):665–73.
50. Mandalakas AM, Ngo K, Alonso Ustero P, Golin R, Anabwani F, Mzileni B, et al. BUTIMBA: Intensifying the Hunt for Child TB in Swaziland through Household Contact Tracing. PLoS One. 2017;12(1):e0169769.
51. Martinez L, Shen Y, Handel A, Chakraburty S, Stein CM, Malone LL, et al. Effectiveness of WHO's pragmatic screening algorithm for child contacts of tuberculosis cases in resource-constrained settings: a prospective cohort study in Uganda. Lancet Respir Med. 2018;6(4):276–86.
52. Maung HMW, Saw S, Isaakidis P, Khogali M, Reid A, Hoa NB, et al. The contribution of a non-governmental organisation's Community Based Tuberculosis Care Programme to case finding in Myanmar: trend over time. Infect Dis Poverty. 2017;6(1):51.

53. Mekonnen A, Collins JM, Aseffa A, Ameni G, Petros B. Prevalence of pulmonary tuberculosis among students in three eastern Ethiopian universities. Int J Tuberc Lung Dis. 2018;22(10):1210–5.
54. Moh R, Badjé A, N'Takpé JB, Kouamé GM, Gabillard D, Ouassa T, et al. Screening for active tuberculosis before isoniazid preventive therapy among HIV-infected West African adults. Int J Tuberc Lung Dis. 2017;21(12):1237-44.
55. Munim IMS, Shewade HD, Jeyashree K, Islam S, Rifat IA, Patwary FK, et al. Financial support to the poor for the detection of smear-negative pulmonary and extra-pulmonary TB in Bangladesh. Int J Tuberc Lung Dis. 2020;24(2):180–8.
56. Myint O, Saw S, Isaakidis P, Khogali M, Reid A, Hoa NB, et al. Active case-finding for tuberculosis by mobile teams in Myanmar: yield and treatment outcomes. Infect Dis Poverty. 2017;6(1):77.
57. Naowarat S, Rojanaworarit C, Surinsak W, Umain K, Ruadreaw D, Yuenprakone S, et al. Tuberculosis case finding: Supplement intensified case finding among acute lower respiratory infection (ALRI) hospitalized patients in Sa Kaeo province, Thailand. J Formos Med Assoc. 2019;118(8):1255–65.
58. Ohene S-A, Bonsu F, Hanson-Nortey NN, Sackey A, Danso S, Afutu F, et al. Yield of tuberculosis among household contacts of tuberculosis patients in Accra, Ghana. Infect Dis Poverty. 2018;7(1):14.
59. Okelloh D, Achola M, Opole J, Ogwang C, Agaya J, Sifuna P, et al. Lessons learned from community-based tuberculosis case-finding in western Kenya. Public Heal Action. 2019;9(2):53–7.
60. Oshi DC, Omeje JC, Oshi SN, Alobu IN, Chukwu NE, Nwokocha C, et al. An evaluation of innovative community-based approaches and systematic tuberculosis screening to improve tuberculosis case detection in Ebonyi State, Nigeria. Int J Mycobacteriology. 2017;6(3):246–52.
61. Putra IWGAE, Kurniasari NMD, Dewi NPEP, Suarjana IK, Duana IMK, Mulyawan IKH, et al. The Implementation of Early Detection in Tuberculosis Contact Investigation to Improve Case Finding. J Epidemiol Glob Health. 2019;9(3):191–7.
62. Sander MS, Laah SN, Titahong CN, Lele C, Kinge T, de Jong BC, et al. Systematic screening for tuberculosis among hospital outpatients in Cameroon: The role of screening and testing algorithms to improve case detection. J Clin Tuberc other Mycobact Dis. 2019;15:100095.
63. Shenoi S V, Moll AP, Brooks RP, Kyriakides T, Andrews L, Kompala T, et al. Integrated Tuberculosis/Human Immunodeficiency Virus Community-Based Case Finding in Rural South Africa: Implications for Tuberculosis Control Efforts. Open Forum Infect Dis. 2017;4(3).
64. Soe KT, Saw S, van Griensven J, Zhou S, Win L, Chinnakali P, et al. International non-governmental organizations' provision of community-based tuberculosis care for hard-to-reach populations in Myanmar, 2013-2014. Infect Dis Poverty. 2017;6(1):69.
65. Tefera F, Barnabee G, Sharma A, Feleke B, Atnafu D, Haymanot N, et al. Evaluation of facility and community-based active

household tuberculosis contact investigation in Ethiopia: a cross-sectional study. BMC Health Serv Res. 2019;19(1):234.
66. Ullah W, Almansour H, Fatima R, Saini B, Khan GM. Engaging Community Pharmacies in Early Detection of Missing Tuberculosis Patients through Public-Private Mix Intervention in Pakistan. Am J Trop Med Hyg. 2020;103(1):221–30.
67. Zawedde-Muyanja S, Nakanwagi A, Dongo JP, Sekadde MP, Nyinoburyo R, Ssentongo G, et al. Decentralisation of child tuberculosis services increases case finding and uptake of preventive therapy in Uganda. Int J Tuberc Lung Dis. 2018;22(11):1314–21.
68. Zhang C, Xia L, Rainey JJ, Li Y, Chen C, Rao Z, et al. Findings from a pilot project to assess the feasibility of active tuberculosis case finding among seniors in rural Sichuan Province, China, 2017. PLoS One. 2019;14(3):e0214761.
69. Abdullahi SA, Smelyanskaya M, John S, Adamu HI, Ubochioma E, Kennedy I, et al. Providing TB and HIV outreach services to internally displaced populations in Northeast Nigeria: Results of a controlled intervention study. PLoS Med. 2020;17(9):e1003218.
70. Bogdanova E, Mariandyshev O, Hinderaker SG, Nikishova E, Kulizhskaya A, Sveshnikova O, et al. Mass screening for active case finding of pulmonary tuberculosis in the Russian Federation: how to save costs. Int J Tuberc Lung Dis. 2019;23(7):830–7.
71. Chen J-O, Qiu Y-B, Rueda ZV, Hou J-L, Lu K-Y, Chen L-P, et al. Role of community-based active case finding in screening tuberculosis in Yunnan province of China. Infect Dis Poverty. 2019;8(1):92.
72. Daftary A, Satyanarayana S, Jha N, Singh M, Mondal S, Vadnais C, et al. Can community pharmacists improve tuberculosis case finding? A mixed methods intervention study in India. BMJ Glob Heal. 2019;4(3):e001417.
73. Datiko DG, Yassin MA, Theobald SJ, Blok L, Suvanand S, Creswell J, et al. Health extension workers improve tuberculosis case finding and treatment outcome in Ethiopia: a large-scale implementation study. BMJ Glob Heal. 2017;2(4):e000390.
74. Datta B, Prakash AK, Ford D, Tanwar PK, Goyal P, Chatterjee P, et al. Comparison of clinical and cost-effectiveness of two strategies using mobile digital x-ray to detect pulmonary tuberculosis in rural India. BMC Public Health. 2019;19(1):99.
75. Gurung SC, Dixit K, Rai B, Caws M, Paudel PR, Dhital R, et al. The role of active case finding in reducing patient incurred catastrophic costs for tuberculosis in Nepal. Infect Dis Poverty. 2019;8(1):99.
76. Hussain H, Mori AT, Khan AJ, Khowaja S, Creswell J, Tylleskar T, et al. The cost-effectiveness of incentive-based active case finding for tuberculosis (TB) control in the private sector Karachi, Pakistan. BMC Health Serv Res. 2019;19(1):690.
77. James R, Khim K, Boudarene L, Yoong J, Phalla C, Saint S, et al. Tuberculosis active case finding in Cambodia: a pragmatic, cost-effectiveness comparison of three implementation models. BMC Infect Dis. 2017;17(1):580.
78. Jaramillo E. The impact of media-based health education on tuberculosis diagnosis in Cali, Colombia. Health Policy Plan. 2001;16(1):68–73.
79. Jose B, Manhica I, Jones J, Mutaquiha C, Zindoga P, Eduardo I, et al. Using community health workers for facility and community

based TB case finding: An evaluation in central Mozambique. PLoS One. 2020;15(7):e0236262.
80. Joshi B, Chinnakali P, Shrestha A, Das M, Kumar AMV, Pant R, et al. Impact of intensified case-finding strategies on childhood TB case registration in Nepal. Public Heal Action. 2015;5(2):93–8.
81. Khan AJ, Khowaja S, Khan FS, Qazi F, Lotia I, Habib A, et al. Engaging the private sector to increase tuberculosis case detection: An impact evaluation study. Lancet Infect Dis. 2012;12:608–16.
82. Khan MA, Munir MA, Anil S, Ahmad M, Walley J, Qadeer E, et al. Structured performance monitoring of TB-care at facility, district and province levels — Pakistan experience. J Pak Med Assoc. 2016;66:418–24.
83. Khatana GH, Haq I, Khan SMS. Effectiveness, acceptance and feasibility of home-based intervention model for tuberculosis contact tracing in Kashmir. J Clin Tuberc other Mycobact Dis. 2019;14:19–25.
84. Lung T, Marks GB, Nhung NV, Anh NT, Hoa NLP, Anh LTN, et al. Household contact investigation for the detection of tuberculosis in Vietnam: economic evaluation of a cluster-randomised trial. Lancet Glob Heal. 2019;7(3):e376–84.
85. Malik AA, Amanullah F, Codlin AJ, Siddiqui S, Jaswal M, Ahmed JF, et al. Improving childhood tuberculosis detection and treatment through facility-based screening in rural Pakistan. Int J Tuberc Lung Dis. 2018;22(8):851–7.
86. McAllister S, Wiem Lestari B, Sujatmiko B, Siregar A, Sihaloho ED, Fathania D, et al. Feasibility of two active case finding approaches for detection of tuberculosis in Bandung City, Indonesia. Public Heal Action. 2017;7(3):206–11.
87. Morishita F, Eang MT, Nishikiori N, Yadav RP. Increased case notification through active case finding of tuberculosis among household and neighbourhood contacts in Cambodia. PLoS One. 2016;11(3):1–14.
88. Morishita F, Garfin AMCG, Lew W, Oh KH, Yadav R-P, Reston JC, et al. Bringing state-of-the-art diagnostics to vulnerable populations: The use of a mobile screening unit in active case finding for tuberculosis in Palawan, the Philippines. PLoS One. 2017;12(2):e0171310.
89. Ohene S-A, Bonsu F, Hanson-Nortey NN, Toonstra A, Sackey A, Lonnroth K, et al. Provider initiated tuberculosis case finding in outpatient departments of health care facilities in Ghana: yield by screening strategy and target group. BMC Infect Dis. 2017;17(1):739.
90. Oshi DC, Chukwu JN, Nwafor CC, Meka AO, Madichie NO, Ogbudebe CL, et al. Does intensified case finding increase tuberculosis case notification among children in resource-poor settings? A report from Nigeria. Int J Mycobacteriology. 2016;5:44–50.
91. Pan D, Lin M, Lan R, Graviss EA, Lin D, Liang D, et al. Tuberculosis Transmission in Households and Classrooms of Adolescent Cases Compared to the Community in China. Int J Environ Res Public Health. 2018;15(12).
92. Reddy K., Ananthakrishan R, Jacob AG, Das M, Isaakidis P, Kumar AMV. Intensified tuberculosis case finding amongst vulnerable com- munities in southern India. Public Heal Action. 2015;5(4):246–8.

93. Saunders MJ, Tovar MA, Collier D, Baldwin MR, Montoya R, Valencia TR, et al. Active and passive case-finding in tuberculosis-affected households in Peru: a 10-year prospective cohort study. Lancet Infect Dis. 2019;19(5):519–28.
94. Shah L, Rojas Pena M, Mori O, Zamudio C, Kaufman JS, Otero L, et al. A pragmatic stepped-wedge cluster randomized trial to evaluate the effectiveness and cost-effectiveness of active case finding for household contacts within a routine tuberculosis program, San Juan de Lurigancho, Lima, Peru. Int J Infect Dis. 2020;100:95–103.
95. Shewade HD, Gupta V, Ghule VH, Nayak S, Satyanarayana S, Dayal R, et al. Impact of Advocacy, Communication, Social Mobilization and Active Case Finding on TB Notification in Jharkhand, India. J Epidemiol Glob Health. 2019;9(4):233–42.
96. Ustero PA, Kay AW, Ngo K, Golin R, Tsabedze B, Mzileni B, et al. School and household tuberculosis contact investigations in Swaziland: Active TB case finding in a high HIV/TB burden setting. PLoS One. 2017;12(6):e0178873.
97. Vo LNQ, Forse RJ, Codlin AJ, Vu TN, Le GT, Do GC, et al. A comparative impact evaluation of two human resource models for community-based active tuberculosis case finding in Ho Chi Minh City, Viet Nam. BMC Public Health. 2020;20(1):934.
98. Vyas A, Creswell J, Codlin AJ, Stevens R, Rao VG, Kumar B, et al. Community-based active case-finding to reach the most vulnerable: tuberculosis in tribal areas of India. Int J Tuberc Lung Dis. 2019;23(6):750–5.
99. Yassin MA, Datiko DG, Tulloch O, Markos P, Aschalew M, Shargie EB, et al. Innovative Community-Based Approaches Doubled Tuberculosis Case Notification and Improve Treatment Outcome in Southern Ethiopia. PLoS One. 2013;8(5):e63174–e63174.
100. Yellappa V, Battaglioli T, Gurum SK, Narayanan D, Van der Stuyft P. Involving private practitioners in the Indian tuberculosis programme: a randomised trial. Trop Med Int Health. 2018;23(5):570–9.
101. Yuen CM, Agaya J, McHembere W, Okelloh D, Achola M, Opole J, et al. Optimizing the efficiency of tuberculosis active case-finding in health facilities and communities. Int J Tuberc Lung Dis. 2019;23(7):844–9.
