# Supplementary material for: Repairing boundaries along pathways to tuberculosis case detection: a qualitative synthesis of intervention designs
Source: Health Res Policy Syst. 2022 Jan 10;20:7. doi: 10.1186/s12961-021-00811-0 (PMC8751340; doi:10.1186/s12961-021-00811-0)
Supplement: Supplementary file 3 — Additional file 3. Data tables. Table S1. Characteristics of included studies. Table S2. Authors’ description of interventions. Table S3. Intervention activities (codes), References. [file 12961_2021_811_MOESM3_ESM.pdf]

Table S1: Characteristics of included studies

Study ID	Intervention dates	Country	TB burden	Rural or urban	Study design	Unit of randomization and size	Participants (age)
Ayles 2013 (1)	1 August 2006 through 31 July 2009	South Africa and Zambia	Notification rate for each community was at least 400 per 100000 per year	Rural and urban	Cluster randomized trial	24 Communities<br>Total population in intervention areas: 962 655	Adults (≥ 18 years of age)
Bello 2017 (2)	March 2010 through January 2012	Malawi	Mean TB cases starting treatment at baseline<br>Arm 1: 112 TB cases/month<br>Arm 2: 124 TB cases/ month	Rural	Cluster randomized trial	6 Clusters<br>Average cluster population size: 209 564	Adults (≥ 12 years of age)
Calligaro 2017 (3)	18 October 2013 to 31 March 2015	SA and Zimbabwe	Total TB incidence per 100 000 population<br>SA: 615<br>Zimbabwe: 199 *	Rural	Individual randomized controlled trial	2261 Individuals were screened<br>875 Screen positive individuals were randomized into two diagnostic groups	Adults (≥ 18 years of age)
Cavalcante 2010 (4)	November 2000 to December 2004	Brazil	TB incidence rate in Rio de Janeiro: 240 per 100000 population	Urban	Cluster randomized trial	8 Neighborhoods<br>Population in study area: 265 000	2147 contacts of 712 TB index cases
Clarke 2005 (5)	1 November 2000 to 31 October 2001	SA	A total of 148 TB cases at baseline	Rural	Cluster randomized trial	211 Farms<br>Intervention farms: 4438/8887 adult population<br>Control farms: 4449/8887 adult population	Adults (≥ 15 years of age)
Corbett 2010 (6)	January 2006 to November 2008	Zimbabwe	Baseline culture-positive TB prevalence: 6.5 per 1000 adults	Urban	Cluster randomized trial	46 Clusters<br>Total population size: 110 432 adults	Adults (≥ 16 years of age)
Datiko 2009 (7)	September 2006 to April 2008	Ethiopia	Total TB incidence: 140 per 100 000 population*	Rural	Cluster randomized trial	51 Kebeles<br>Total population size: 296 811	Whole population (all ages)
Fairall 2005 (8)	2003	SA	Total TB incidence: 615 per 100 000 population*	Rural and urban	Cluster randomized trial	40 Primary care clinics	1999 patients with cough or difficulty breathing
Fox 2018 (9)	October 2010 through June 2015	Vietnam	In 2015, there were 102,676 registered cases of TB in the country	Rural and urban	Cluster randomized trial	70 Districts<br>Total population size: 15 849 559	25707 household contacts (all ages) of 10964 index patients
Hanrahan 2019 (10)	July 2016 to January 2018	SA	TB prevalence was estimated at 300/100,000	> 60% Rural	Cluster randomized trial	56 Public-sector primary care clinics in 2 districts	Contacts (all ages) of 3655 TB index patients
Jenum 2018 (11)	November 2006 to September 2010	India	Total TB incidence: 193 per 100 000 population*	Rural and urban	Cluster randomized trial	Villages or subsections of towns<br>Total neonates: 4382	BCG vaccinated neonates within 15 days of birth
Khan 2016	June 2012 to May	Pakistan	Total TB incidence:	Rural	Cluster	318 Neighbourhoods	All ages

(12)	2013		263 per 100 000 population*		randomized trial	Population of 3.19 million	
Marks 2019 (13)	March 2014 through February 2018	Vietnam	Estimated prevalence of culture-proven TB was 350 per 100,000 population	Not reported	Cluster randomized trial	120 Subcommunes<br>Average cluster population of approximately 1000 persons ≥ 15 years of age	Adults (≥ 15 years of age)
Miller 2010 (14)	August 2005 to March 2006	Brazil	Estimated TB incidence of 565 per 100 000 population	Urban	Cluster randomized trial	14 Neighbourhoods<br>Estimated population size: 58 587	All ages in households with at least one member ≥ 18 years of age
Moyo 2012 (15)	2005 to 2008	SA	Overall TB incidence was 1442 per 100 000 population	Rural	Individual randomized trial	N = 4786 infants enrolled	BCG vaccinated infants within 2 weeks of birth
Shargie 2006 (16)	May 2003 through April 2004	Ethiopia	Estimated incidence of new smear positive TB in 2003 in country was 155 per 100000 population	Rural	Cluster randomized trial	32 Communities<br>Average cluster size of 11 000 people	All ages
Talukder 2012 (17)	2008 to 2009	Bangladesh	Child TB prevalence of 52/100 000 children	Not reported	Cluster randomized trial	36 Microscopy centres<br>Total number of children evaluated: 3460	Children
* Baseline data not reported. TB incidence from WHO TB country profile data 2019 (18)							

Table S2: Authors' description of interventions

Study ID	Arm	Authors' description of intervention(s)
Ayles 2013 (1)	1	Strengthened tuberculosis-HIV programme at the clinic
	2	Community level enhanced case finding<br>& Strengthened tuberculosis-HIV programme at the clinic
	3	Household level TB-HIV care<br>& Strengthened tuberculosis-HIV programme at the clinic
	4	Community level enhanced case finding<br>& Household level TB-HIV care<br>& Strengthened tuberculosis-HIV programme at the clinic
Bello 2017 (2)	1	Engaging informal healthcare providers in an integrated TB and HIV community intervention
	2	No intervention
Calligaro 2017 (3)	1	Community-based intensified case finding (novel diagnostic group)
	2	Community-based intensified case finding (routine diagnostic group)
Cavalcante 2010 (4)	1	DOTS with added intensive screening of household contacts
	2	Standard DOTS
Clarke 2005 (5)	1	Lay Health Worker intervention
	2	No intervention
Corbett 2010 (6)	1	Active case finding strategy
	2	Active case finding strategy
Datiko 2009 (7)	1	Health education
	2	Health education and training of health extension workers on how to identify TB suspects, how to collect, label, store and transport sputum specimens and administer DOT
Fairall 2010 (8)	1	Educational outreach to primary care nurses to increase TB case detection and improve respiratory care (Practical Approach to Lung Health in South Africa)
Fox 2018 (9)	1	Active case finding among household contacts of patients with TB

	2	Passive case finding
Hanrahan 2019 (10)	1	Active case finding: contact tracing, household-based & Active case finding: contact tracing, incentive-based
	2	Standard care in SA
Jenum 2018 (11)	1	Active surveillance
	2	Passive surveillance
	2	No intervention
Khan 2016 (12)	1	Simple chest camp
	2	Infotainment chest camp
Marks 2019 (13)	1	Active community wide screening
	2	No community wide screening
Miller 2010 (14)	1	Active TB case finding
	2	Enhanced case finding strategy
Moyo 2012 (15)	1	TB case finding for vaccine trials
	2	No intervention
Shargie 2006 (16)	1	Community outreach case finding
	2	No intervention
Talukder 2012 (17)	1	Provision of child TB guidelines, training and logistics support to staff of microscopy centres
	2	No intervention

Table S3: Intervention activities (codes)

ACTIVITY SET 1: ACTIVITIES TO ENHANCE CARE-SEEKING PATHWAYS TO HEALTHCARE SERVICES; N = 8 studies

Activity set 1A: Activities to enhance the care-seeking pathway **to general health services; n = 6 studies** (2,7,9,11,14,17)

Examples of **general health services** included services at established health facilities, e.g. health posts (7), microscopy centres (17) and informal healthcare provider services (2)

Activities	Illustrative quotes
a) TB health promotion activities	"TB and HIV community awareness meetings" (Bello 2017) (2)<br>"Health education sessions at health posts." "Advised people to come to health post if they had productive cough more than 2 weeks" (Datiko 2009) (7)<br>"Contacts were given written information about tuberculosis" (Fox 2018) (9)<br>"Parental education about TB symptoms" (Jenum 2018) (11)<br>"Distribution of an educational pamphlet" (Miller 2010) (14)<br>"Health education sessions at health centres and community meetings" (Talukder 2012) (17)
b) Service promotion/invitation activities	"Sensitisation of local leaders to TB and HIV activities in the community" (Bello 2017) (2)<br>"People were told about community-based TB treatment" (Datiko 2009) (7)<br>"TB index patients were asked to bring symptomatic child contacts, 5-14 years of age, to the clinic" (Talukder 2012) (17)

Activity set 1B: Activities to enhance the TB care-seeking pathway **to TB diagnostic services; n = 2 studies** (6,16)

These services invited people with TB symptoms and provided TB diagnostic services for people perceiving themselves to have TB symptoms:

"Individuals reporting symptoms to staff waiting by the van provided sputum samples, and could report symptoms and obtain containers on behalf of other individuals within their household." (6)

"Every month, before the outreach day, the promoters went around the villages for 3-4 consecutive days visiting houses, distributing TB leaflets, and discussing the possible symptoms of TB with individuals, households, and community groups. They also promoted messages about TB in schools and popular gatherings in the intervention areas. They encouraged symptomatic TB suspects to visit the outreach team or a nearby health facility if preferred." (16)

Activities	Illustrative quotes
a) TB health promotion activities	"Leafleting" (Corbett 2010) (6)<br>"Every month, before the outreach day, the promoters went around the villages distributing TB leaflets, and discussing the possible symptoms of TB with individuals, households, and community groups." (Shargie 2006) (16)
b) Service promotion/invitation activities	"Used a loudspeaker to publicise leafleting and services provided by one team of three lay field workers." (Corbett 2010) (6)<br>"They encouraged symptomatic TB suspects to visit the outreach team or a nearby health facility if preferred" (Shargie 2006) (16)
c) Improved availability of TB	"The mobile van was located in each cluster for 5 days per intervention round from 9 am to 4 pm, including

diagnostic services	Saturdays.” (Corbett 2010) (6)<br>“The health workers made monthly outreach visits to each intervention kebele. Symptomatic TB suspects submitted the first spot sputum specimen at the outreach site.” (Shargie 2006) (16)
d) Inviting all people in target group regardless of symptoms:<br>**Dedicated TB screening services (see ACTIVITY SET 2)**	“For the open access/fast track and community sputum collection points, sputum was collected from any individual who wanted to provide it regardless of symptoms.” (Ayles 2013) (1)<br>“Contacts were invited” (Fox 2018) (9)<br>“10 paper vouchers to distribute to close contacts (e.g., household members, friends, or coworkers). The vouchers provided information on when to present to the local clinic for TB screening” (Hanrahan 2019) (10)<br>“Adult TB cases were requested to bring all children < 5 years of age (symptomatic and asymptomatic) to the clinic” (Talukder 2012) (17)<br>“Passers-by were encouraged to participate” (Calligaro 2017) (3)<br>“The campaign included announcements at public places like mosques, markets, and schools with invitations to the chest camp and hanging posters with the invitation for the community.”<br>(Khan 2016) (12)

**ACTIVITY SET 2: ACTIVITIES TO ENHANCE SCREENING UPTAKE AT DEDICATED TB SCREENING SERVICES; N = 14 studies**		
**Activity set 2A:** Activities to enhance screening uptake **at dedicated TB screening services with open invitation; n = 6 studies**<br>**TB contacts (n = 3 studies** (9,10,17)**); Whole populations (n = 3 studies** (1,3,12))		
**Activities**	**Target group**	**Illustrative quotes**
a) TB health promotion activities	TB contacts	“Contacts were given written information about tuberculosis” (Fox 2018) (9)<br>“Health education sessions at health centres and community meetings” (Talukder 2012) (17)
	Whole populations	“Community mobilisation and promotion” (Ayles 2013) (1)<br>“The infotainment chest camp consisted of a package of community awareness and advocacy about symptoms, diagnosis, and treatment of TB in the form of an infotainment event (entertaining program giving information about TB) for all the members of the community 1 day before the camp.” (Khan 2016) (12)
b) Service promotion/invitation activities	TB contacts	“Contacts were invited to attend screening at the district clinic” (Fox 2018) (9)<br>“Consenting index patients were given 10 paper vouchers to distribute to close contacts (e.g., household members, friends, or coworkers). The vouchers provided information on when to present to the local clinic for TB screening” (Hanrahan 2019) (10)<br>“Adult TB cases were requested to bring all children < 5 years of age (symptomatic and asymptomatic) to the clinic” (Talukder 2012) (17)
	Whole populations	“Information about the availability of this service (sputum collection points) was included in the community mobilisation messaging.” (Ayles 2013) (1)<br>“The vehicle was parked at these locations, and passers-by were encouraged to participate using a loudspeaker, by advertising banners displayed next to the vehicle, and by local advertising at schools, churches, supermarkets, and social clubs.” (Calligaro 2017) (3)<br>“The simple chest camp consisted of a simple advocacy campaign in the community before the camp. The campaign included announcements at public places like mosques, markets, and schools with invitations to the chest camp and hanging posters with the invitation for the community.” (Khan 2016) (12)
c) Improved availability of TB screening service	Whole populations	“Sputum collection points” (Ayles 2013) (1)<br>“Vehicle parked at these locations” (Calligaro 2017) (3)<br>“Chest camp” (Khan 2016) (12)
d) Incentives	TB contacts	“Conditional cash transfer at each scheduled visit to cover travel expenses” (Fox 2018) (9)<br>“In the incentive-based contact-tracing arm, both index patients and their contacts were given monetary incentives if the contact presented to the clinic for screening.” (Hanrahan 2019) (10)
e) Bypass access barriers; TB screening personally offered to target group members at their homes<br>**(see Activity set 2B)**	TB contacts	“At the household, all contacts present were offered TB screening” (Hanrahan 2019) (10)<br>“These counsellors visited the household of all newly diagnosed TB patients” (Ayles 2013) (1)
	Whole populations	“TB screening of all permanent farm dweller families.” (Clarke 2005) (5)<br>“Door-to-door enquiry” (Corbett 2010) (6)<br>“We conducted a house-to-house survey of the entire population” (Marks 2019) (13)<br>“All households with at least one member aged ≥ 18 years received a 7-question TB symptom survey after providing verbal consent” (Miller 2010) (14)

	BCG vaccinated infants	"At a bimonthly home visit, a study field worker enquired about TB-related symptoms and recent TB exposure, recorded the weight of the child (calibrated spring balance) on a WHO gender-specific growth chart" (Jenum 2018) (11)<br>"Home visits every 3 months for questionnaire-based screening for TB symptoms and contacts." (Moyo 2012) (15)
**Activity set 2B:** Activities to enhance screening uptake **when TB screening is personally offered to target group members at their homes; n = 9 studies**<br>**TB contacts: n = 3 studies** (1,4,10); **Whole populations: n = 4 studies** (5,6,13,14); **BCG vaccinated infants: n = 2 studies** (11,15)		
**Activities**	**Target group**	**Illustrative quotes**
a) TB health promotion activities	TB contacts	"In addition the counsellors provided TB/HIV education" (Ayles 2013) (1)
	Whole populations	"Leaflets explained the study rationale and stressed the benefits to family and friends of early diagnosis of tuberculosis, and the important role of HIV-negative tuberculosis in persistence of transmission." (Corbett 2010) (6)
	BCG vaccinated infants	"All parents/caretakers were educated about TB-related symptoms and encouraged to contact a study worker or visit the case verification ward (transportation free of cost), if their children developed symptoms suggestive of TB or were exposed to a TB case" (Jenum 2018) (11)
b) Repeat visits	TB contacts	"These counsellors visited the household of all newly diagnosed TB patients at least 3 times during the patient's treatment" (Ayles 2013) (1)
	Whole populations	"LHWs conducted monthly weighing and TB screening of all permanent farm dweller families." (Clarke 2005) (5)<br>"Door-to-door enquiry for chronic cough and leafleting was done by two teams of three lay field workers. Households were visited up to three times per round between 9 am and 4 pm, including one weekend visit, until at least one member was present." (Corbett 2010) (6)
	BCG vaccinated infants	"At a bimonthly home visit, a study field worker enquired about TB-related symptoms and recent TB exposure, recorded the weight of the child (calibrated spring balance) on a WHO gender-specific growth chart" (Jenum 2018) (11)<br>"Home visits every 3 months for questionnaire-based screening for TB symptoms and contacts." (Moyo 2012) (15)

**ACTIVITY SET 3: IDENTIFICATION OF PRESUMPTIVE TB AT GENERAL HEALTH SERVICES; N = 5 studies** (2,7,8,10,17)	
**Activities**	**Illustrative quotes**
a) Activities to improve sensitivity of health workers to the possibility of TB<br>• TB training<br>• Diagnostic tools to help classify patients identified with respiratory symptoms	"Training of informal health care providers to recognize individuals with symptoms suggestive of TB" (Bello 2017) (2)<br>"Training of health workers on symptoms and transmission of TB and how to identify TB suspects" (Datiko 2009) (7)<br>"The study recruited 1999 patients with cough or difficult breathing attending the trial clinics. The intervention was based on the PALSA syndromic guideline for management of adult respiratory diseases, which classified patients into diagnostic and treatment categories according to their symptoms and signs." (Fairall 2010) (8)<br>"Health care workers were trained to weigh children accurately, assess for severe malnutrition using weight-for-age tables and perform the Mantoux tuberculin skin test." (Talukder 2012) (17)
b) Systematic screening of all people seeking care at the facility	"Screening all those attending the clinic and presenting with TB symptoms using WHO's recommended four-symptom screen, often at point of clinic registration" (Hanrahan 2019) (8)
**ACTIVITY SET 4: ACTIVITIES TO IMPROVE ACCESS TO TB DIAGNOSTIC SERVICES AFTER IDENTIFICATION OF PRESUMPTIVE TB; N = 12 studies**	
**Activity set 4A:** Activities to improve access to TB diagnostic services after identification of presumptive TB **at general health services; n = 3 studies** (17)	
**Activities**	**Illustrative quotes**
• Referral for diagnosis at a health facility<br>• Provision of diagnostic tools to health workers<br>• Training of health workers to use diagnostic tools	"Training of informal health care providers to encourage individuals with suggestive symptoms to attend a local health facility for diagnosis and clinical management. Some providers were also trained to collect TB sputum specimens." (Bello 2017) (10)<br>"Training of health workers on how to collect, label, store and transport sputum specimens" (Datiko 2009) (2,7,17)<br>"TB guidelines, training and logistics support. The Keith Edwards Child TB Score Chart was used as the main diagnostic tool." (Talukder 2012) (2)

**Activity set 4B:** Activities to improve access to TB diagnostic services after identification of presumptive TB **at dedicated TB screening services with open invitation; n = 3 studies** (7)		
• Sputum collection at outreach site<br>• Mobile laboratory	Whole populations	"For the open access/fast track and community sputum collection points, sputum was collected from any individual who wanted to provide it regardless of symptoms. Sputum collection points were set up in the community in a rotating manner so that the whole community was covered. The ECF intervention was developed according to 2 principles: that every person in the community should be able to deliver a sputum sample within 30-minute walk of their home at least 3 times per year, and that sputum smear results would be available within 48 hours. (Ayles 2013) (17)<br>"In South Africa, the diagnostic tests were done at the point-of-contact at the mobile van using a generator to power the Xpert machine, whereas in Zimbabwe, screened and eligible participants were transported to Mabvuku Clinic and the investigations were done there" (Calligaro 2017) (1,3,12)<br>"A mobile laboratory was set up at all the chest camps with all the equipment and reagents available." (Khan 2016) (1)
**Activity set 4C:** Activities to improve access to TB diagnostic services after identification of presumptive TB **at target group members' homes; n = 8 studies** (3)		
• Referral for diagnosis at a health facility	TB contacts	"TB symptom screening (using a standardized symptom checklist with referral for sputum examination for any individuals found to be positive on the screen)" (Ayles 2013) (12)
	Whole populations	"They referred individuals with two or more signs and symptoms suggestive of TB to the clinic for further investigation." (Clarke 2005) (1,5,6,10,11,13–15)
	BCG vaccinated infants	"If referral criteria were present, the field worker encouraged, repeatedly if needed, that the parent/caretaker brings the child to the case verification ward" (Jenum 2018) (1)<br>"All suspects were referred to a study research ward for evaluation for TB disease." (Moyo 2012) (5)
• Sputum collection at home	TB contacts	"Screen positive contacts not already on TB treatment had sputum collected for Xpert MTB/RIF testing." (Hanrahan 2019) (11)
	Whole populations	"Specimen containers and instructions were left if symptoms were volunteered for any household members." (Corbett 2010) (15)<br>"Sputum samples, collected from the participants who consented and were able to provide a sample, were transferred to the laboratory." (Marks 2019) (10)<br>"Those reporting cough for ≥ 3 weeks were asked to provide a sputum specimen. A second sputum collection pot was left with the household and collected the following day." (Miller 2010) (6)

**ACTIVITY SET 5: HIV COUNSELING AND TESTING AND TB SCREENING OFFERED TO PEOPLE LIVING WITH HIV; N = 2 studies** (13)	
**Activities**	**Illustrative quotes**
• HIV counseling and testing added to TB screening<br>• HIV counseling and testing for all people seeking care at clinics and linkage to HIV care<br>• TB screening offered to all people with HIV infection	"Additionally, all communities had a strengthened tuberculosis–HIV programme implemented at the clinic, in line with WHO policy for collaborative tuberculosis–HIV activities, which involved strengthening of laboratory diagnosis for tuberculosis, augmentation of the tuberculosis registration system, HIV testing offered to all patients with referral for HIV care and antiretroviral therapy, increased tuberculosis screening for individuals with HIV infection, and provision of isoniazid preventive therapy." "In addition the counsellors provided TB/HIV education, TB symptom screening (using a standardized symptom checklist with referral for sputum examination for any individuals found to be positive on the screen), HIV counselling and testing, linkage to HIV care, adherence support and isoniazid preventive therapy to all consenting household members." (Ayles 2013) (14)<br>"After obtaining informed consent, participants were counselled, and their HIV status confirmed by fingerprick tests using two commercially available assays. The screening protocol depended on HIV status: we consecutively enrolled HIV-positive patients aged 18 years or older with at least one symptom of tuberculosis according to predefined WHO criteria but enrolled all adult HIV-positive patients irrespective of symptoms in line with the WHO recommendation to screen all HIV-positive individuals for tuberculosis. Patients who declined HIV testing were screened as though they were HIV-positive." (Calligaro 2017) (1,3)
**ACTIVITY SET 6: HEALTH SYSTEM STRENGTHENING TO SUPPORT INTERVENTION ACTIVITIES AND TO SUPPORT THE POTENTIAL INCREASE IN TB CASE DETECTION AS A RESULT OF INTERVENTION ACTIVITIES; N = 4 studies** (1)	
**Activities**	**Illustrative quotes**
• Training of lay health workers to conduct screening	"Training took place at eight central venues, in groups of six to 10 participants. Farmers provided transport for LHWs. LHWs conducted monthly weighing and TB screening of all permanent farm dweller families." (Clarke 2005) (3)

• Improved storage and transport of sputum specimens	"In both intervention groups, specimens were transported and processed centrally" (Corbett 2010) (1,5–7)<br>"An ice box was used to keep the sputum specimens in the health post and during their transportation on foot to diagnostic units." (Datiko 2009) (5)
• Laboratory strengthening<br>• Health information system augmentation	"Additionally, all communities had a strengthened tuberculosis–HIV programme implemented at the clinic, in line with WHO policy for collaborative tuberculosis–HIV activities, which involved strengthening of laboratory diagnosis for tuberculosis, augmentation of the tuberculosis registration system" (Ayles 2013) (6)<br>"We trained laboratory technicians" (Datiko 2009) (7)
• Improved TB treatment support<br>• Improved TB preventive therapy support	"Adherence support and isoniazid preventive therapy" (Ayles 2013) (1)

References

1. Ayles H, Muyoyeta M, Du Toit E, Schaap A, Floyd S, Simwinga M, et al. Effect of household and community interventions on the burden of tuberculosis in southern Africa: The ZAMSTAR community-randomised trial. Lancet. 2013;382:1183–94.
2. Bello G, Faragher B, Sanudi L, Namakhoma I, Banda H, Malmborg R, et al. The effect of engaging unpaid informal providers on case detection and treatment initiation rates for TB and HIV in rural Malawi (Triage Plus): A cluster randomised health system intervention trial. PLoS One. 2017;12(9):e0183312.
3. Calligaro GL, Zijenah LS, Peter JG, Theron G, Buser V, McNerney R, et al. Effect of new tuberculosis diagnostic technologies on community-based intensified case finding: a multicentre randomised controlled trial. Lancet Infect Dis. 2017;17(4):441–50.
4. Cavalcante SC, Durovni B, Barnes GL, Souza FBA, Silva RF, Barroso PF, et al. Community-randomized trial of enhanced DOTS for tuberculosis control in Rio de Janeiro, Brazil. Int J Tuberc Lung Dis. 2010;14(2):203–9.
5. Clarke M, Dick J, Zwarenstein M, Lombard CJ, Diwan VK. Lay health worker intervention with choice of DOT superior to standard TB care for farm dwellers in South Africa: A cluster randomised control trial. Int J Tuberc Lung Dis. 2005;9(6):673–9.
6. Corbett EL, Bandason T, Duong T, Dauya E, Makamure B, Churchyard GJ, et al. Comparison of two active case-finding strategies for community-based diagnosis of symptomatic smear-positive tuberculosis and control of infectious tuberculosis in Harare, Zimbabwe (DETECTB): A cluster-randomised trial. Lancet. 2010;376(9748):1244–53.
7. Datiko DG, Lindtjørn B. Health extension workers improve tuberculosis case detection and treatment success in southern Ethiopia: A community randomized trial. PLoS One. 2009;4(5):e5443.
8. Fairall LR, Zwarenstein M, Bateman ED, Bachmann M, Lombard C, Majara BP, et al. Effect of educational outreach to nurses on tuberculosis detection and primary care of respiratory illness: pragmatic cluster randomised controlled trial. BMJ. 2005;331(October):750–4.
9. Fox GJ, Nhung N V., Sy DN, Hoa NLP, Anh LTN, Anh NT, et al. Household-Contact Investigation

for Detection of Tuberculosis in Vietnam. N Engl J Med. 2018;378(3):221–9.

10. Hanrahan CF, Nonyane BAS, Mmolawa L, West NS, Siwelana T, Lebina L, et al. Contact tracing versus facility-based screening for active TB case finding in rural South Africa: A pragmatic cluster-randomized trial (Kharitode TB). PLoS Med. 2019;16(4):e1002796.
11. Jenum S, Selvam S, Jesuraj N, Ritz C, Hesseling AC, Cardenas V, et al. Incidence of tuberculosis and the influence of surveillance strategy on tuberculosis case-finding and all-cause mortality: a cluster randomised trial in Indian neonates vaccinated with BCG. BMJ open Respir Res. 2018;5(1):e000304.
12. Khan MA, Anil S, Ahmed M, Athar A, Ghafoor A, Brouwer M. Active Case Finding of Tuberculosis: randomized Evaluation of Simple and Infotainment Chest Camps. Ann Glob Heal. 2016;82(5):813-18.
13. Marks GB, Nguyen N V., Nguyen PTB, Nguyen T-A, Nguyen HB, Tran KH, et al. Community-wide Screening for Tuberculosis in a High-Prevalence Setting. N Engl J Med. 2019;381(14):1347–57.
14. Miller AC, Golub JE, Cavalcante SC, Durovni B, Moulton LH, Fonseca Z, et al. Controlled trial of active tuberculosis case finding in a Brazilian favela. Int J Tuberc Lung Dis. 2010;14(6):720–6.
15. Moyo S, Verver S, Hawkridge A, Geiter L, Hatherill M, Workman L, et al. Tuberculosis case finding for vaccine trials in young children in high-incidence settings: A randomised trial. Int J Tuberc Lung Dis. 2012;16(2):185–91.
16. Shargie EB, Mørkve O, Lindtjørn B. Tuberculosis case-finding through a village outreach programme in a rural setting in southern Ethiopia: Community randomized trial. Bull World Health Organ. 2006;84(2):112–9.
17. Talukder K, Salim MAH, Jerin I, Sharmin F, Talukder MQK, Marais BJ, et al. Intervention to increase detection of childhood tuberculosis in Bangladesh. Int J Tuberc Lung Dis. 2012;16(1):70–5.
18. World Health Organization (WHO). TB burden estimates and country-reported TB data. 2019. Available from: https://www.who.int/tb/country/data/profiles/en/ Accessed 13 Apr 2020.
